# Prevalence of Pre‐Clinical Obesity Among US Adults Receiving Medical and Surgical Obesity Treatment

**DOI:** 10.1002/oby.70171

**Published:** 2026-05-20

**Authors:** Jason M. Samuels, Emma Holler, Matthew D. Spann, C. Joe Northup, Tarik K. Yuce

**Affiliations:** ^1^ Department of General Surgery, Section of Surgical Sciences Vanderbilt University Medical Center Nashville Tennessee USA; ^2^ Surgical Outcomes and Quality Improvement Center (SOQIC), Department of Surgery, Indiana University School of Medicine Indianapolis Indiana USA

**Keywords:** bariatric surgery, clinical obesity, lancet commission, obesity medications, pre‐clinical obesity

## Abstract

**Objective:**

We sought to determine how the proportion of adults historically receiving obesity interventions would be classified under the recently proposed “pre‐clinical” and “clinical” obesity classifications and the impact of this change on safety and outcomes.

**Methods:**

A retrospective analysis of adults with BMI ≥ 35 who received bariatric surgery (BS) or antiobesity medications (AOMs) between 2017 and 2023 was conducted using the Merative MarketScan database. Patients had ≥ 6‐month continuous enrollment before treatment. Clinical obesity was defined as presence of ≥ 1 obesity‐related condition and pre‐clinical obesity defined as absence of such. Treatment‐related complications were identified within 90 days of treatment initiation. Multivariable logistic regression was used to compare the odds of treatment‐related complications by obesity classification.

**Results:**

A total of 120,499 individuals (46.1%) underwent BS and 140,909 (53.9%) received AOMs. Among BS patients, 12.7% (*n* = 15,334) had pre‐clinical obesity. Of AOM users, 38.1% (*n* = 53,633) had pre‐clinical obesity. Clinical obesity patients were older with a higher comorbidity burden compared to pre‐clinical obesity. Clinical obesity was associated with higher odds of post‐treatment complications (AOM: aOR 1.10, BS: aOR 1.39, both *p* < 0.001).

**Conclusions:**

This novel framework would reclassify a significant proportion of patients as having pre‐clinical obesity. Use of these definitions to determine treatment eligibility could potentially delay intervention and shift care to older, sicker populations.

## Study Importance

- What is already known?
  ○ The recent *Lancet Diabetes & Endocrinology* Commission on obesity guidelines has the potential to dramatically reclassify patients with obesity.
- What does this study add?
  ○ Our study finds that 1/10th to 1/3rd of patients who have historically undergone bariatric surgery and medical treatment for obesity, respectively, would have been classified as having pre‐clinical obesity and could potentially have care withheld by payers as a result.

## 1 Introduction

The recent *Lancet Diabetes and Endocrinology* Commission on “Definition and Diagnostic Criteria of Clinical Obesity” proposed a redefinition of obesity as a disease characterized by excess adiposity, shifting focus away from body mass index (BMI) as the sole diagnostic criterion [1]. This new framework introduces two categories: “pre‐clinical obesity” (excess adiposity without complications) and “clinical obesity” (excess adiposity with one or more complications), with the aim of refining diagnosis and guiding treatment decisions [1]. However, the practical utility and implications of implementing this classification remain uncertain.

Although the Lancet Commission aims to standardize obesity diagnosis, several concerns have been raised regarding both the criteria and their potential consequences. First, the criteria excluded certain conditions found to associate closely with obesity such as type 2 diabetes and dyslipidemia. Additionally, the requirement for direct adiposity measurements (e.g., with waist circumference or dual‐energy x‐ray absorptiometry [DXA]) is complicated by the lack of validated standards across sex, race, and life‐span. A recent *JAMA* study demonstrated 98% concordance between BMI and excess adiposity measured by DXA, raising questions about whether this revised framework offers meaningful diagnostic improvement over existing methods [2]. Moreover, professional organizations including the Obesity Medicine Association have cautioned that payer adoption of these definitions could restrict access to obesity treatments or delay care until patients have advanced disease (i.e., clinical obesity) [3]. Such delays may have clinically important consequences: research indicates that postponing bariatric surgery leads to increased health care costs and diminished effectiveness, with longer delays correlating with worse outcomes [4]. Furthermore, early‐onset obesity is associated with a higher risk of premature mortality and chronic diseases in adulthood [5]. If payers adopt the proposed classification and restrict treatment to those with clinical obesity, many individuals currently receiving treatment could become ineligible, potentially leading to delayed care. This shift may lead to worse health outcomes, greater lifetime medical expenditures, and diminished returns from treatment.

To date, no studies have examined how this proposed classification might influence eligibility for obesity interventions such as bariatric surgery or antiobesity medications (AOMs), including glucagon‐like peptide‐1 receptor agonists (GLP‐1 RAs). In addition, differences in short‐term treatment‐related complications between patients meeting criteria for pre‐clinical versus clinical obesity remain poorly described. To address these gaps, we conducted a population‐level analysis to (1) estimate the proportion of patients receiving bariatric surgery or AOMs who would be reclassified as having pre‐clinical obesity under the proposed criteria, (2) characterize the most common obesity‐associated conditions among patients meeting the new definition of clinical obesity and those not included in the definition (i.e., type 2 diabetes), and (3) compare treatment‐related complications by obesity classification.

## 2 Methods

We conducted a retrospective cohort study using the Merative MarketScan Commercial claims database to identify adults (≥ 18 years) with BMI ≥ 35 kg/m^2^ who received bariatric surgery or AOMs between January 1, 2017, and December 31, 2023. This study was deemed not human subjects research by the Indiana University Institutional Review Board because it used a deidentified dataset; therefore, IRB review was waived. This study adheres to the reporting standards described in the Strengthening the Reporting of Observational Studies in Epidemiology (STROBE) guidelines [6].

Patients were included if they had BMI ≥ 35, evidence of qualifying obesity treatment (bariatric surgery or AOM prescription), and ≥ 6 months of continuous insurance enrollment prior to the treatment date. Based upon the finding by Aryee et al. that excess adiposity defined by BMI ≥ 35 has 98% concordance with DXA‐measured excess adiposity, we used BMI ≥ 35 as the sole measure of excess adiposity in our study [2]. An ICD code for BMI ≥ 35 kg/m^2^ in the pre‐treatment period was also required (Table S1) for all patients. The index date was defined as the earliest date of a qualifying intervention. For patients receiving AOMs, the index date corresponded to the first qualifying pharmacy claim with no prior AOM use in the preceding 6 months to define new initiation. Patients with a diagnosis of type 2 diabetes during the pre‐index period were excluded from the AOM cohort to ensure the AOMs were used for weight loss. Patients in the surgery cohort were not excluded if they had a diagnosis of type 2 diabetes.

Bariatric procedures were identified using CPT codes (Table S2). AOM use was defined as at least one pharmacy claim for any of the following medications: bupropion/naltrexone, exenatide, liraglutide, lorcaserin, orlistat, phentermine/topiramate, semaglutide, setmelanotide, or tirzepatide (Table S4).

The Lancet Commission defines clinical obesity as excess adiposity accompanied by evidence of organ dysfunction and/or limitations in daily activities [1]. Because direct measures of adiposity (e.g., DXA scans) are not available in claims data, we assumed that all individuals with BMI ≥ 35 met the threshold for excess adiposity. To operationalize the Commission's definition, we classified patients as having clinical obesity if they had at least one obesity‐related complication documented via ICD‐10‐CM codes during the 6‐month pre‐index period. Qualifying complications included cardiovascular disease, renal (chronic kidney disease stage ≥ 3) or liver disease (e.g., metabolic‐associated steatotic liver disease), metabolic disorders (e.g., hypertension, dyslipidemia), mechanical complications (e.g., osteoarthritis, obstructive sleep apnea), functional limitations (e.g., impairments in activities of daily living), and other obesity‐related diagnoses (see Table S3 for full list). Consistent with the Commission's criteria, type 2 diabetes was considered a qualifying condition only when accompanied by mixed hyperlipidemia. Patients without any of these conditions were classified as having pre‐clinical obesity.

Treatment‐related complications were assessed in the subset of patients who had at least 3 months of continuous enrollment after initial treatment. Potential medication‐ and surgery‐related complications were identified based on the presence of one or more relevant ICD codes recorded within 90 days after initial treatment (Tables S4 and S5). Medication‐related complications included gastrointestinal conditions such as gastroesophageal reflux disease (GERD), abdominal pain, diarrhea or constipation, nausea and vomiting, volume depletion, gastroparesis, or bowel obstruction. Hepatobiliary complications included drug‐induced pancreatitis, cholelithiasis, and cholecystitis. Additional medication‐related complications encompassed acute kidney injury, renal and ureteral calculi, hypoglycemia, volume depletion disorders, and allergic reactions. Surgery‐related complications included infections (e.g., surgical site infections, sepsis, peritonitis) and gastrointestinal issues (e.g., nausea and vomiting, nutrient malabsorption, Mallory‐Weiss syndrome, gastrojejunostomy ulcers), as well as anastomotic leak, perforation, incisional hernia, seroma, and venous thromboembolism.

Descriptive analyses were performed to estimate the proportion of patients in each treatment group (bariatric surgery or AOM) who met criteria for pre‐clinical versus clinical obesity. Patient characteristics including age, sex, geographic region, insurance type, Charlson Comorbidity Index (CCI) [7], and other relevant demographic and clinical variables were compared between groups. Missing data, if present, were handled by creating a separate missing category. Categorical variables were compared using chi‐square tests and continuous variables were compared using two‐sample *t*‐tests. The association between obesity classification and treatment‐related complications was assessed using a multivariable logistic regression model including obesity class, age, sex, and U.S. geographic region. Comorbidity variables were not included in the regression as they are what distinguish pre‐clinical obesity from clinical obesity. *p* values < 0.05 were considered statistically significant and all analyses were completed using R version 4.4.1 (R Foundation for Statistical Computing, Vienna, Austria).

## 3 Results

### 3.1 Participants

**FIGURE 1:**
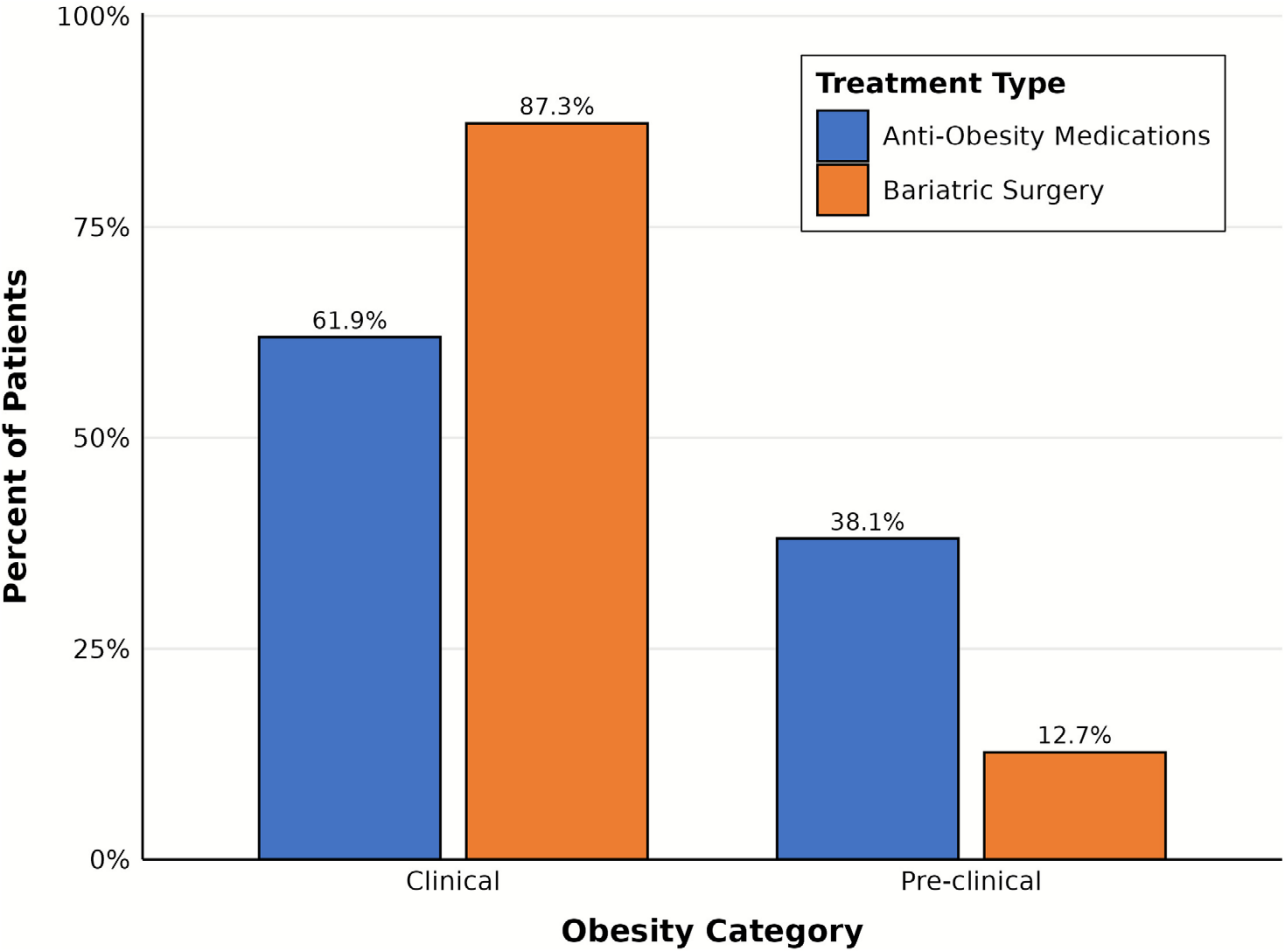
Proportion of patients with clinical versus pre‐clinical obesity by obesity treatment type. [Color figure can be viewed at wileyonlinelibrary.com]

Of 261,408 adults who received obesity treatments, 120,499 (46.1%) underwent bariatric surgery and 140,909 (53.9%) utilized AOMs. The surgical cohort included 15,334 (12.7%) patients with pre‐clinical obesity, while the AOM cohort included 53,633 (38.1%) patients with pre‐clinical obesity (Figure 1). Age and sex distributions varied by obesity classification. Patients with pre‐clinical obesity were significantly younger than those with clinical obesity in both treatment groups (mean age: 41.1 vs. 46.3 years for AOM users and 36.7 vs. 43.7 years for surgical patients; both *p* < 0.001) (Table 1). Women represented a higher proportion of pre‐clinical obesity patients in both the AOM cohort (86% vs. 74%) and surgical cohort (92% vs. 78%) compared to those with clinical obesity (both *p* < 0.001). Patients with clinical obesity also had higher BMI at the time of treatment in the AOM cohort. Among AOM users, 76% of those with clinical obesity had BMI ≥ 40 kg/m^2^ compared to 65% in the pre‐clinical group. This pattern was reversed in the surgical cohort, where a slightly higher percentage of patients with pre‐clinical obesity (99.2%) had BMI ≥ 40 kg/m^2^ compared to those with clinical obesity (98.7%) (*p* < 0.001).

**TABLE 1:** Patient characteristics by treatment type and obesity classification.

Variable	Obesity medication (*N* = 140,909)		*p*	Bariatric surgery (*N* = 120,499)		*p*	Total (*N* = 261,408)
	Pre‐clinical obesity (*N* = 53,633)	Clinical obesity (*N* = 87,276)		Pre‐clinical obesity (*N* = 15,334)	Clinical obesity (*N* = 105,165)		
Age, years, mean (SD)	41.1 (10.2)	46.3 (10.4)	< 0.001	36.7 (9.3)	43.7 (10.2)	< 0.001	43.6 (10.5)
Sex			< 0.001			< 0.001	
Female	46,323 (86%)	64,875 (74%)		14,037 (92%)	82,361 (78%)		207,596 (79%)
Male	7310 (14%)	22,401 (26%)		1297 (8.5%)	22,804 (22%)		53,812 (21%)
BMI category			< 0.001			< 0.001	
35–39.9	18,828 (35%)	20,992 (24%)		118 (0.8%)	1391 (1.3%)		41,329 (16%)
40+	34,805 (65%)	66,284 (76%)		15,216 (99%)	103,774 (99%)		220,079 (84%)
U.S. region			< 0.001			< 0.001	
North central	11,955 (22%)	19,147 (22%)		3392 (22%)	22,112 (21%)		56,606 (22%)
Northeast	8766 (16%)	13,639 (16%)		2249 (15%)	17,875 (17%)		42,529 (16%)
South	28,807 (54%)	47,936 (55%)		7823 (51%)	53,402 (51%)		137,968 (53%)
Unknown	40 (< 0.1%)	54 (< 0.1%)		13 (< 0.1%)	101 (< 0.1%)		208 (< 0.1%)
West	4065 (7.6%)	6500 (7.4%)		1857 (12%)	11,675 (11%)		24,097 (9.2%)
Insurance type			< 0.001			< 0.001	
CDHP	8323 (16%)	13,092 (15%)		1885 (12%)	12,552 (12%)		35,852 (14%)
EPO	189 (0.4%)	369 (0.4%)		176 (1.1%)	1270 (1.2%)		2004 (0.8%)
HDHP	6516 (12%)	9843 (11%)		1667 (11%)	10,072 (9.6%)		28,098 (11%)
HMO	5019 (9.4%)	8799 (10%)		1850 (12%)	12,122 (12%)		27,790 (11%)
Other	1295 (2.4%)	2076 (2.4%)		318 (2.1%)	2628 (2.5%)		6317 (2.4%)
POS (w/ or w/o capitation)	3603 (6.7%)	5922 (6.8%)		1093 (7.1%)	8451 (8.0%)		19,069 (7.3%)
PPO	27,440 (51%)	45,350 (52%)		7946 (52%)	55,564 (53%)		136,300 (52%)
Unknown	1248 (2.3%)	1825 (2.1%)		399 (2.6%)	2506 (2.4%)		5978 (2.3%)
Bariatric procedure			NA			< 0.001	
Duodenal switch	—	—		133 (0.9%)	1342 (1.3%)		1475 (0.6%)
Gastric band	—	—		217 (1.4%)	871 (0.8%)		1088 (0.4%)
RYGB	—	—		3374 (22%)	28,293 (27%)		31,667 (12.1%)
Sleeve	—	—		11,610 (76%)	74,659 (71%)		86,269 (33.0%)
None	—	—		0 (0%)	0 (0%)		140,909 (53.9%)
Obesity medication			< 0.001			NA	
Bupropion hydrochloride/naltrexone hydrochloride	4523 (8.4%)	6448 (7.4%)		—	—		10,971 (4.2%)
Exenatide	116 (0.2%)	296 (0.3%)		—	—		412 (0.2%)
Liraglutide	14,097 (26%)	23,314 (27%)		—	—		37,411 (14.3%)
Lorcaserin hydrochloride	1502 (2.8%)	2485 (2.8%)		—	—		3987 (1.5%)
Orlistat	127 (0.2%)	204 (0.2%)		—	—		331 (0.1%)
Phentermine hydrochloride/topiramate	1835 (3.4%)	2399 (2.7%)		—	—		4234 (1.6%)
Semaglutide	28,043 (52%)	46,620 (53%)		—	—		74,663 (28.6%)
Setmelanotide	2 (< 0.1%)	1 (< 0.1%)		—	—		3 (0.0%)
Tirzepatide	3388 (6.3%)	5509 (6.3%)		—	—		8897 (3.4%)
None	0 (0%)	0 (0%)		—	—		120,499 (46.1%)
Charlson comorbidity score			< 0.001			< 0.001	
0	36,650 (68%)	38,285 (44%)		9724 (63%)	29,166 (28%)		113,825 (44%)
1	12,694 (24%)	28,613 (33%)		4233 (28%)	34,447 (33%)		79,987 (31%)
2	3308 (6.2%)	13,401 (15%)		1023 (6.7%)	21,926 (21%)		39,658 (15%)
3+	981 (1.8%)	6977 (8.0%)		354 (2.3%)	19,626 (19%)		27,938 (11%)

Abbreviations: CDHP, Consumer‐Driven Health Plan; EPO, Exclusive Provider Organization Plan; HDHP, High‐Deductible Health Plan; HMO, Health Maintenance Organization Plan; POS, Point of Service Plan; PPO, Preferred Provider Organization Plan; RYGB, Roux en Y gastric bypass.

Comorbidity burden, measured using the Charlson Comorbidity Index (CCI), a general measure of disease burden not specific to obesity, was significantly greater among those with clinical obesity. Most pre‐clinical obesity patients had a CCI score of 0 (68% of AOM users and 63% of surgical patients) compared to much lower proportions among clinical obesity patients (44% of AOM users and 28% of surgical patients *p* < 0.001). In contrast, 19% of clinical obesity patients undergoing surgery had a CCI ≥ 3 compared to just 1.8% in the pre‐clinical group.

Among patients with clinical obesity, the most prevalent obesity‐related conditions were hypertension (*n* = 125,479; 65%), obstructive sleep apnea (86,775; 45%), arthritis (49,376; 26%), metabolic‐associated steatotic liver disease (30,734; 16%), and type 2 diabetes without mixed hyperlipidemia (24,580; 18%) (Table 2).

**TABLE 2:** Prevalence of obesity‐related conditions among patients with clinical obesity.

Obesity‐related condition	Obesity medication (*N* = 87,276)	Bariatric surgery (*N* = 105,165)	Total (*N* = 192,441)
Venous thromboembolism	1312 (1.5%)	1716 (1.6%)	3028 (1.6%)
Metabolic‐associated steatotic liver disease	6239 (7.1%)	24,495 (23%)	30,734 (16%)
Activities of daily living impairment	150 (0.2%)	256 (0.2%)	406 (0.2%)
Incontinence	2413 (2.8%)	3698 (3.5%)	6111 (3.2%)
Chronic kidney disease (stage 3+)	911 (1.0%)	1686 (1.6%)	2597 (1.3%)
Congestive heart failure	1823 (2.1%)	3180 (3.0%)	5003 (2.6%)
Atrial fibrillation	386 (0.4%)	348 (0.3%)	734 (0.4%)
Pulmonary hypertension	503 (0.6%)	1007 (1.0%)	1510 (0.8%)
Idiopathic intracranial hypertension	645 (0.7%)	809 (0.8%)	1454 (0.8%)
Obstructive sleep apnea	26,112 (30%)	60,663 (58%)	86,775 (45%)
Arthritis	22,685 (26%)	26,691 (25%)	49,376 (26%)
Female infertility	10,656 (12%)	10,750 (10%)	21,406 (11%)
Male infertility	3586 (4.1%)	2168 (2.1%)	5754 (3.0%)
Lymphedema	816 (0.9%)	825 (0.8%)	1641 (0.9%)
Hypertension	54,754 (63%)	70,725 (67%)	125,479 (65%)
Type 2 diabetes with mixed hyperlipidemia^a^	0 (0%)	8827 (8.4%)	8827 (4.6%)
Type 2 diabetes without mixed hyperlipidemia^a^	0 (0%)	24,580 (23%)	24,580 (13%)
Obesity hypoventilation syndrome	479 (0.5%)	2184 (2.1%)	2663 (1.4%)

^a^ Patients with type 2 diabetes were excluded from the obesity medication cohort.

### 3.2 Treatment‐Related Complications

A total of 15,184 AOM users (10.8%) and 14,352 bariatric surgery patients (11.9%) were excluded from the complications analysis due to insufficient follow‐up (< 90 days of continuous enrollment after treatment). Among the remaining patients, treatment‐related complications occurred in 19% of AOM users and 33% of surgical patients. The most common complications among AOM users were GERD (7.6%), abdominal pain (5.8%), and diarrhea or constipation (5.6%). In surgical patients, the most frequently observed complications were nutrient malabsorption (13%), nausea or vomiting (13%), and peritonitis (7.0%). Complication rates were significantly higher among patients with clinical obesity compared to those with pre‐clinical obesity among both AOM users (19.8% vs. 14.7%, *p* < 0.001) and surgical patients (32.9% vs. 30.5%, *p* < 0.001) (Tables 3 and 4). After adjusting for age, sex, and U.S. geographic region, patients with clinical obesity had higher odds of experiencing complications compared to patients with pre‐clinical obesity; the odds were 1.10 times greater among surgical patients (OR 1.10, 95% CI 1.06–1.15, *p* < 0.001), and 1.39 times greater among AOM users (OR 1.39, 95% CI 1.34–1.44, *p* < 0.001).

**TABLE 3:** Frequency of medication‐related complications within 90 days of treatment initiation.

Medication‐related complications	Pre‐clinical obesity (*N* = 47,763)	Clinical obesity (*N* = 77,962)	Total (*N* = 125,725)	*p*
Any complication	7572 (16%)	15,727 (20%)	23,299 (19%)	< 0.001
Gastroesophageal reflux disease	2891 (6.1%)	6618 (8.5%)	9509 (7.6%)	< 0.001
Abdominal pain	2365 (5.0%)	4902 (6.3%)	7267 (5.8%)	< 0.001
Diarrhea/constipation	2552 (5.3%)	4458 (5.7%)	7010 (5.6%)	0.005
Nausea/vomiting	1331 (2.8%)	2307 (3.0%)	3638 (2.9%)	0.077
Volume depletion disorders	498 (1.0%)	1759 (2.3%)	2257 (1.8%)	< 0.001
Urinary complications	379 (0.8%)	954 (1.2%)	1333 (1.1%)	< 0.001
Gastroparesis/bowel obstruction	40 (< 0.1%)	115 (0.1%)	155 (0.1%)	0.002
Drug‐induced pancreatitis	34 (< 0.1%)	74 (< 0.1%)	108 (< 0.1%)	0.20
Hypoglycemia	163 (0.3%)	332 (0.4%)	495 (0.4%)	0.020
Cholelithiasis/cholecystitis	273 (0.6%)	607 (0.8%)	880 (0.7%)	< 0.001
Allergic reactions	70 (0.1%)	122 (0.2%)	192 (0.2%)	0.70
Injection site reactions	23 (< 0.1%)	41 (< 0.1%)	64 (< 0.1%)	0.70
Acute kidney injury	31 (< 0.1%)	276 (0.4%)	307 (0.2%)	< 0.001

*Note: N* = 15,184 (10.8%) patients without 90 days of continuous enrollment after treatment were excluded.

**TABLE 4:** Frequency of surgery‐related complications within 90 days of operation.

Surgery‐related complications	Pre‐clinical obesity (*N* = 13,452)	Clinical obesity (*N* = 92,695)	Total (*N* = 106,147)	*p*
Any complication	4157 (31%)	30,765 (33%)	34,922 (33%)	< 0.001
Nutrient malabsorption	1483 (11%)	12,608 (14%)	14,091 (13%)	< 0.001
Nausea/vomiting	1833 (14%)	11,464 (12%)	13,297 (13%)	< 0.001
Peritonitis	751 (5.6%)	6697 (7.2%)	7448 (7.0%)	< 0.001
Bariatric complications	695 (5.2%)	3962 (4.3%)	4657 (4.4%)	< 0.001
Surgical site infection	230 (1.7%)	2485 (2.7%)	2715 (2.6%)	< 0.001
Bowel obstruction	164 (1.2%)	1342 (1.4%)	1506 (1.4%)	0.036
Incisional hernia	96 (0.7%)	870 (0.9%)	966 (0.9%)	0.010
Sepsis	80 (0.6%)	758 (0.8%)	838 (0.8%)	0.006
Venous thromboembolism	44 (0.3%)	716 (0.8%)	760 (0.7%)	< 0.001
Gastrojejunal ulcer	59 (0.4%)	564 (0.6%)	623 (0.6%)	0.016
Seroma	4 (< 0.1%)	56 (< 0.1%)	60 (< 0.1%)	0.20
Perforation	26 (0.2%)	237 (0.3%)	263 (0.2%)	0.20
Mallory‐Weiss syndrome	9 (< 0.1%)	47 (< 0.1%)	56 (< 0.1%)	0.40
Anastomotic leak	186 (1.4%)	1532 (1.7%)	1718 (1.6%)	0.020

*Note: N* = 14,352 (11.9%) surgical patients without 90 days of continuous enrollment after treatment were excluded.

## 4 Discussion

In this population‐based analysis, we found that nearly one‐quarter of patients, all of whom had BMI ≥ 35 kg/m^2^, who historically received obesity treatments would not meet criteria for “clinical obesity” under the definitions put forward by the Lancet Commission. These individuals, lacking documented obesity‐related complications, would be reclassified as having “pre‐clinical obesity” and potentially become ineligible for treatment under the proposed framework. Patients who had ICD diagnosis codes meeting the Commission's definition of clinical obesity, when compared to those who did not, were older and had a higher comorbidity burden, consistent with the expected progression of disease severity over time.

Our findings suggest that payer adoption of clinical/pre‐clinical obesity classification as a coverage criterion could significantly reduce patient eligibility for evidence‐based treatments. This is especially relevant for newer AOMs such as GLP‐1 RAs, which have demonstrated significant weight loss and metabolic benefits but remain underutilized; recent estimates suggest only 2.3% of patients with obesity are currently receiving these medications [8, 9]. Several factors likely contribute to this limited uptake including medication supply challenges, barriers to prescribing by primary care providers, and insurance coverage limitatons [10, 11]. Access to bariatric surgery, despite its long‐established safety and effectiveness, has also been limited due to similar challenges, including insurance exclusions, preoperative requirements, and perceptions around surgical risk [12, 13]. Further restricting access to medical or surgical interventions by requiring patients to meet a clinical obesity threshold may exacerbate existing disparities and delays in care.

In addition to reduced access, delayed initiation of treatment may shift the population receiving therapy toward older individuals with more advanced disease. Prior studies have shown that older age at the time of bariatric surgery is associated with increased perioperative risk, diminished weight loss, and lower rates of remission of obesity‐related conditions such as type 2 diabetes and hypertension [14, 15, 16]. Limited data exist regarding how age impacts the effectiveness and tolerability of AOMs; but given the concern with lean body mass loss with these agents, their use in an older population may carry greater risk for sarcopenia and bone density loss. Early intervention may be more clinically beneficial, and modeling studies have demonstrated cost‐effectiveness and improved quality‐adjusted life years with earlier access to treatment [4].

Our study raises important concerns about the potential risks associated with delaying treatment until the development of obesity‐related conditions. Compared to patients with pre‐clinical obesity, those with clinical obesity had a significantly higher risk of treatment‐related complications following both AOM initiation and bariatric surgery. Previous studies reported that increasing age and male sex are associated with greater odds of gastrointestinal adverse events, both minor and major, following GLP‐1 RA use, as was the presence of comorbidities such as chronic kidney disease and heart failure [17, 18]. Studies have also found associations beween age, sex, and pre‐existing comorbidities and greater odds of complications following bariatric surgery [15, 19, 20]. These findings are consistent with our observation that patients with clinical obesity, who were generally older and more medically complex, experienced more frequent treatment‐related complications. Adoption of the proposed obesity classification as a criterion for treatment eligibility may inadvertently shift access to more advanced therapies toward higher‐risk patients, thereby worsening the safety profile of these interventions.

This study has several limitations. First, the MarketScan database does not include direct measures of adiposity (e.g., waist circumference, body composition). However, we assumed that individuals with BMI ≥ 35 had excess adiposity, which is supported by evidence demonstrating that BMI reflects excess adiposity in approximately 98% of cases [2]. Second, as of October 2022, the American Society for Metabolic and Bariatric Surgery adopted BMI ≥ 35 as new criteria for surgery, regardless of associated conditions [21]. As we include patients who underwent surgery prior to this change from the previous BMI cutoff of ≥ 40, our lower percentage of patients in the surgical cohort with pre‐clinical obesity may reflect this limitation, with patients prior to the change not able to access surgery absent associated comorbidities.

Third, the dataset includes only individuals with commercial insurance and may not reflect the experiences of those with public insurance or those lacking coverage. Fourth, due to limitations in the database structure, we were unable to definitively attribute potential complications to specific treatment episodes. As a result, we assumed that complications occurring within the relevant time frame were related to the treatment, which may overestimate the incidence of these events. Finally, many insurance companies already limit access to care by refusing coverage for medical or surgical weight loss options, regardless of BMI or other measures of adiposity, and our study is unable to determine how adoption of these definitions may change coverage decisions by providers currently not covering obesity treatments.

## 5 Conclusion

While moving beyond BMI represents conceptual progress in obesity medicine, our findings suggest that restricting treatment to patients with “clinical obesity” could exclude a substantial proportion of patients currently receiving evidence‐based therapies, particularly those initiating AOMs. Such policies may delay treatment until comorbidities emerge, shifting treatment toward older, more medically complex patients and potentially reducing effectiveness while increasing short‐term risk and long‐term health care costs. These findings underscore the need for careful consideration of how evolving definitions of obesity are operationalized in clinical and payer decision‐making to avoid unintended consequences that limit access to care and widen disparities in obesity treatment. Finally, an actionable classification system will require clear, standardized criteria, including explicit specification of relevant comorbidities and corresponding ICD codes, to ensure consistent assessment of disease severity.

## Funding

This work was supported by the National Institute of Diabetes and Digestive and Kidney Diseases, K23DK143312, and National Center for Advancing Translational Sciences, K12TR004415.

## Conflicts of Interest

The authors declare no conflicts of interest.

## Supporting information


**Table S1:** ICD codes used to identify BMI 35+.
**Table S2:** Bariatric surgery CPT codes.
**Table S3:** ICD‐10 codes for obesity‐related conditions.
**Table S4:** ICD‐10 codes for medication‐related complications.
**Table S5:** ICD‐10 codes for surgery‐related complications.

## Data Availability

The data that support the findings of this study are available from Merative MarketScan. Restrictions apply to the availability of these data, which were used under license for this study. Data are available from the author(s) with the permission of Merative MarketScan.
