# Supplementary material for: Prevalence of Pre‐Clinical Obesity Among US Adults Receiving Medical and Surgical Obesity Treatment
Source: Obesity (Silver Spring). 2026 May 20;34(7):1493–501. doi: 10.1002/oby.70171 (PMC13306137; doi:10.1002/oby.70171)
Supplement: Supplementary file 1 — Table S1: ICD codes used to identify BMI 35+. Table S2: Bariatric surgery CPT codes. Table S3: ICD‐10 codes for obesity‐related conditions. Table S4: ICD‐10 codes for medication‐related complications. Table S5: ICD‐10 codes for surgery‐related complications. [file OBY-34-1493-s001.docx]

**Appendix Table 1. ICD codes used to identify BMI 35+**

| **ICD-10 Code** | **Description** |
| --- | --- |
| E6601 | Morbid (severe) obesity due to excess calories |
| E66812 | Obesity, class 2 |
| E66813 | Obesity, class 3 |
| Z6834-Z6839 | BMI 35.0-39.9 |
| Z684 | Body mass index [BMI] 40 or greater, adult |

**Appendix Table 2. Bariatric surgery CPT codes**

| **CPT** | **CPT Description** |
| --- | --- |
| 43644 | Laparoscopic RYGB (proximal) |
| 43645 | Laparoscopic RYGB (distal) |
| 43770 | Laparoscopic adjustable gastric band and port implantation |
| 43775 | Laparoscopic Sleeve Gastrectomy |
| 43842 | Vertical banded gastroplasty (VGB) |
| 43843 | Adjustable gastric band (AGB); Gastric restrictive procedure, without gastric bypass, for morbid obesity; other than vertical-banded gastroplasty |
| 43845 | BPD/DS; Gastric restrictive procedure, with partial gastrectomy, pylorus-preserving duodenoileostomy (50 to 100 cm common channel) to limit absorption |
| 43846 | RYGB (proximal) |
| 43847 | RYGB (distal) |

**Appendix Table 3. ICD-10 codes for obesity-related conditions**

| **Condition** | **ICD-10 Code** | **Description** |
| --- | --- | --- |
| **Venous thromboembolism** | I260 | Pulmonary embolism with acute cor pulmonale |
|  | I2601 | Septic pulmonary embolism with acute cor pulmonale |
|  | I2602 | Saddle embolus of pulmonary artery with acute cor pulmonale |
|  | I2609 | Other pulmonary embolism with acute cor pulmonale |
|  | I269 | Pulmonary embolism without acute cor pulmonale |
|  | I2690 | Septic pulmonary embolism without acute cor pulmonale |
|  | I2692 | Saddle embolus of pulmonary artery without acute cor pulmonale |
|  | I2699 | Other pulmonary embolism without acute cor pulmonale |
|  | I821 | Acute embolism and thrombosis of vena cava |
|  | I822 | Acute embolism and thrombosis of renal vein |
|  | I823 | Acute embolism and thrombosis of iliac vein |
|  | I8240 | Acute embolism and thrombosis of unspecified deep veins of lower extremity |
|  | I82401 | Acute embolism and thrombosis of right deep veins of lower extremity |
|  | I82402 | Acute embolism and thrombosis of left deep veins of lower extremity |
|  | I8250 | Chronic embolism and thrombosis of unspecified deep veins of lower extremity |
|  | I82501 | Chronic embolism and thrombosis of right deep veins of lower extremity |
|  | I82502 | Chronic embolism and thrombosis of left deep veins of lower extremity |
|  | I826 | Acute embolism and thrombosis of deep veins of upper extremity |
|  | I82621 | Chronic embolism and thrombosis of superficial veins of upper extremity |
|  | I82622 | Chronic embolism and thrombosis of deep veins of upper extremity |
|  | I82623 | Chronic embolism and thrombosis of superficial veins of lower extremity |
|  | I82624 | Chronic embolism and thrombosis of deep veins of lower extremity |
|  | I82625 | Chronic embolism and thrombosis of unspecified veins of upper extremity |
|  | I82626 | Chronic embolism and thrombosis of unspecified veins of lower extremity |
|  | I82629 | Chronic embolism and thrombosis of unspecified veins |
|  | I827 | Chronic embolism and thrombosis of veins of upper extremity |
|  | I828 | Acute embolism and thrombosis of other specified veins |
|  | I829 | Acute embolism and thrombosis of unspecified veins |
|  | I82A1 | Acute embolism and thrombosis of other specified veins |
|  | I82A19 | acute embolism and thrombosis of unspecified axillary vein |
|  | I82B1 | Acute embolism and thrombosis of subclavian vein |
|  | I82C1 | Acute embolism and thrombosis of internal jugular vein |
|  | I870 | Post-thrombotic syndrome |
| **Metabolic-associated steatotic liver disease** | K760 | NAFLD with fibrosis (use additional code for fibrosis, if applicable) |
|  | K7460 | Unspecified cirrhosis of liver (use additional code for NAFLD/NASH, if documented) |
|  | K7469 | Other cirrhosis of liver (use additional code for NAFLD/NASH, if documented) |
|  | C220 | Liver cell carcinoma (use additional code for NAFLD/NASH, if documented) |
|  | K7689 | Other specified diseases of liver (use additional code for NAFLD/NASH, if documented) |
| **Inability to maintain Activities of Daily Living** | Z740 | Need for assistance with personal care (including bathing, dressing, feeding, toileting, etc.). |
| **Mixed hyperlipidemia** | E782 | Mixed hyperlipidemia |
|  | E781 & E785 | Hyperlipidemia and hypertriglyceridemia |
| **Incontinence** | N393 | Involuntary leakage during physical exertion |
|  | N3941 | Involuntary leakage typically preceded by a sudden, strong urge to void. |
|  | N3946 | Combination of stress and urge incontinence. |
|  | N39490 | Overflow incontinence |
|  | N39498 | Atypical incontinence presentations not classified under the above categories. |
|  | R32 | Incontinence not further specified. |
| **Chronic kidney disease, Stage 3+** | N183 | Chronic kidney disease, Stage 3 (moderate reduction in GFR, 30-59 mL/min) |
|  | N184 | Chronic kidney disease, Stage 4 (severe reduction in GFR, 15-29 mL/min) |
|  | N185 | Chronic kidney disease, Stage 5 (kidney failure, GFR <15 mL/min) |
|  | N186 | End-stage renal disease (requiring chronic dialysis or transplant) |
| **Congestive heart failure** | I110 | Hypertensive heart disease with heart failure. |
|  | I130 | Hypertensive heart and chronic kidney disease with heart failure and stage 1 through stage 4 chronic kidney disease, or unspecified chronic kidney disease |
|  | I132 | Hypertensive heart and chronic kidney disease with heart failure and with stage 5 chronic kidney disease, or end stage renal disease |
|  | I255 | Ischemic cardiomyopathy |
|  | I420 | Dilated cardiomyopathy |
|  | I428 | Other cardiomyopathies |
|  | I429 | Cardiomyopathy, unspecified |
|  | I43 | Cardiomyopathy in diseases classified elsewhere |
|  | I501 | Left ventricular failure, unspecified |
|  | I5020 | Unspecified systolic (congestive) heart failure |
|  | I5021 | Chronic systolic heart failure (reduced ejection fraction). |
|  | I5022 | Chronic systolic heart failure, acute exacerbation. |
|  | I5023 | Acute-on-chronic systolic heart failure. |
|  | I5030 | Unspecified diastolic (congestive) heart failure |
|  | I5031 | Chronic diastolic heart failure (preserved ejection fraction). |
|  | I5032 | Chronic diastolic heart failure, acute exacerbation. |
|  | I5033 | Acute-on-chronic diastolic heart failure. |
|  | I5040 | Unspecified combined systolic (congestive) and diastolic (congestive) heart failure |
|  | I5041 | Combined systolic/diastolic heart failure, chronic. |
|  | I5042 | Combined systolic/diastolic heart failure, acute exacerbation. |
|  | I5043 | Acute-on-chronic combined heart failure. |
|  | I508 | Other heart failure |
|  | I50810 | Chronic right heart failure. |
|  | I50820 | Chronic failure of both ventricles. |
|  | I509 | CHF, unspecified. |
| **Atrial fibrillation** | I481 | Persistent atrial fibrillation |
|  | I4820 | Chronic/persistent AF (continuous for ≥7 days or requiring intervention). |
|  | I4821 | Long-standing AF where rhythm control is no longer pursued. |
| **Pulmonary hypertension** | I270 | Idiopathic pulmonary arterial hypertension (PAH) (no identifiable cause). |
|  | I272 | PH due to underlying conditions (e.g., lung disease, heart disease). |
|  | I2720 | PH without specification of cause or type. |
|  | I2721 | Post-capillary PH (e.g., due to left ventricular failure, mitral valve disease). |
|  | I2722 | PH secondary to chronic obstructive lung disease (COPD) or interstitial lung disease. |
|  | I2723 | PH due to chronic pulmonary embolism. |
|  | I2724 | PH linked to congenital cardiac shunts (e.g., atrial septal defect). |
|  | I2729 | PH caused by medications (e.g., appetite suppressants, cocaine). Example: T45.515A (adverse effect of appetite suppressants). |
|  | I2781 | Acute cor pulmonale (right heart failure secondary to PH). |
|  | I2782 | Chronic right heart failure due to PH. |
| **Idiopathic intracranial hypertension (Pseudotumor Cerebri)** | G932 | Elevated intracranial pressure without an identifiable cause; commonly associated with obesity and may present with headache. |
| **Obstructive Sleep Apnea** | G4733 | Obstructive Sleep Apnea (OSA), Unspecified |
|  | G4736 | Sleep-Related Hypoventilation, Unspecified |
| **Osteoarthritis** | M160 | Bilateral primary osteoarthritis of hip |
|  | M161 | Unilateral primary osteoarthritis of hip |
|  | M165 | Unilateral post-traumatic osteoarthritis of hip |
|  | M166 | Other bilateral secondary osteoarthritis of hip |
|  | M167 | Other unilateral secondary osteoarthritis of hip |
|  | M169 | Osteoarthritis of hip, unspecified |
|  | M170 | Bilateral primary osteoarthritis of knee |
|  | M171 | Unilateral primary osteoarthritis of knee |
|  | M174 | Other bilateral secondary osteoarthritis of knee |
|  | M175 | Other unilateral secondary osteoarthritis of knee |
|  | M179 | Osteoarthritis of knee, unspecified |
|  | M190 | Primary osteoarthritis of other joints |
|  | M192 | Secondary osteoarthritis of other joints |
|  | M199 | Osteoarthritis, unspecified site |
|  | M2555 | Pain in hip |
|  | M2556 | Pain in knee |
| **PCOS & female infertility** | E282 | Endocrine disorder in women characterized by ovarian dysfunction, menstrual irregularities, and hyperandrogenism. |
|  | N91 | Abnormal menstrual cycle patterns (including amenorrhea and oligomenorrhea) frequently encountered in PCOS patients. |
|  | N97 | Infertility often related to anovulatory cycles associated with PCOS. |
| **Male infertility** | E230 | Hypogonadism due to pituitary/hypothalamic dysfunction (e.g., tumors, radiation). |
|  | E233 | Hypogonadism due to hypothalamic disorders (e.g., Kallmann syndrome). |
|  | E291 | Androgen deficiency due to testicular failure (e.g., low testosterone). |
|  | E298 | Other testicular dysfunction |
|  | E299 | Hypogonadism, unspecified cause. |
|  | N460 | Azoospermia |
|  | N461 | Oligospermia |
|  | N468 | Other male infertility |
|  | N469 | Male infertility, unspecified |
| **Secondary lymphedema** | I890 | Lymphedema acquired due to lymphatic injury from surgery, radiation, trauma, infection, or other causes. |
| **Type 2 diabetes mellitus** | E11 | Type 2 diabetes mellitus |
|  | Z7984 | Long-term (current) use of oral hypoglycemic drugs |
| **Hypertension** | I10 | Primary (essential) hypertension without mention of heart, kidney, or other organ involvement. |
|  | I119 | Hypertensive heart disease without heart failure. |
|  | I120 | Hypertensive chronic kidney disease with stage 5 chronic kidney disease or end stage renal disease |
|  | I129 | Chronic kidney disease attributed to hypertension without heart failure. |
|  | I131 | Hypertensive heart and chronic kidney disease without heart failure |
|  | I150 | Renovascular hypertension |
|  | I151 | Hypertension secondary to other renal disorders |
|  | I152 | Hypertension secondary to endocrine disorders |
|  | I158 | Other secondary hypertension |
|  | I159 | Secondary hypertension, unspecified |
| **Obesity hypoventilation syndrome** | E662 | Obesity Hypoventilation Syndrome (Pickwickian Syndrome) |
| Note: ICD codes were identified using partial string matching. Any codes containing the character sequence shown (including all descendant/child codes that share that prefix) were included. | | |

**Appendix Table 4. ICD-10 codes for medication-related complications**

| **Category** | **ICD-10 Code** | **Description** |
| --- | --- | --- |
| **Abdominal pain** | G8918 | Other acute postprocedural pain |
|  | G8928 | Other chronic postprocedural pain |
|  | R109 | Unspecified abdominal pain |
|  | R1011 | Right upper quadrant pain |
|  | R1012 | Left upper quadrant pain |
|  | R1013 | Epigastric pain |
|  | R1014 | Left lower quadrant pain |
|  | R1033 | Periumbilical pain |
|  | R1030 | Lower abdominal pain, unspecified |
|  | R1084 | Generalized abdominal pain |
|  | R109 | Unspecified abdominal pain |
| **Nausea/vomiting** | R110 | Nausea |
|  | R1110 | Vomiting, unspecified |
|  | R112 | Nausea with vomiting, unspecified |
| **Diarrhea/**  **constipation** | R197 | Diarrhea, unspecified |
|  | K590 | Constipation |
| **Gastroparesis** | K3184 | Gastroparesis |
| **Cholelithiasis/cholecystitis** | K80 | Cholelithiasis |
|  | K81 | Cholecystitis |
|  | K830 | Cholangitis |
| **Drug-induced pancreatitis** | K85 | Acute pancreatitis |
| **Volume depletion disorders** | E860 | Dehydration |
|  | E8351 | Hypocalcemia |
|  | E8352 | Hypercalcemia |
|  | E871 | Hypo-osmolality and hyponatremia |
|  | E872 | Acidosis |
|  | E873 | Alkalosis |
|  | E874 | Mixed disorder of acid-base balance |
|  | E875 | Hyperkalemia |
|  | E876 | Hypokalemia |
|  | E878 | Other disorders of electrolyte and fluid balance, not elsewhere classified |
| **Acute kidney injury** | N17 | Acute kidney failure |
| **Hypoglycemia** | E160 | Drug-induced hypoglycemia without coma |
|  | E161 | Other hypoglycemia |
| **GERD** | K219 | Gastro-esophageal reflux disease without esophagitis |
|  | K3189 | Other diseases of stomach and duodenum |
| **Urinary complications** | N201 | Calculus of ureter |
|  | N200 | Calculus of kidney |
| **Allergic reactions** | T887XXA | Unspecified adverse effect of drug or medicament, initial encounter |
|  | T887XXD | Unspecified adverse effect of drug or medicament, subsequent encounter |
|  | T887XXS | Unspecified adverse effect of drug or medicament, sequela |
| **Injection site reactions** | L270 | Generalized skin eruption due to drugs and medicaments taken internally |
|  | T782XXA | Anaphylactic shock, unspecified, initial encounter |
|  | T8089XA | Other complications following infusion, transfusion and therapeutic injection, initial encounter |
| Note: ICD codes were identified using partial string matching. Any codes containing the character sequence shown (including all descendant/child codes that share that prefix) were included. | | |

**Appendix Table 5. ICD-10 codes for surgery-related complications**

| **Category** | **ICD-10 Code** | **Description** |
| --- | --- | --- |
| **General bariatric complications** | K95 | Complications of bariatric procedures |
|  | K9501 | Infection due to gastric band procedure |
|  | K9509 | Other complications of gastric band procedure |
|  | K9581 | Infection due to other bariatric procedure |
|  | K9589 | Other complications of other bariatric procedure |
| **Peritonitis** | K63 | Other diseases of intestine (e.g., abscess, perforation, fistula) |
|  | K65 | Peritonitis (acute, chronic, or unspecified) |
|  | K66 | Other disorders of peritoneum |
|  | K67 | Disorders of peritoneum in infectious diseases classified elsewhere |
|  | K68 | Other specified disorders of peritoneum |
|  | K91850 | Pouchitis |
|  | K91858 | Other complications of intestinal pouch |
|  | K9289 | Other specified diseases of the digestive system |
|  | K929 | Disease of digestive system, unspecified |
| **Surgical site infections** | T814 | Infection following a procedure |
|  | T8140 | Infection following a procedure, unspecified |
|  | T8141 | Infection following a procedure, superficial incisional surgical site |
|  | T8142 | Infection following a procedure, deep incisional surgical site |
|  | T8143 | Infection following a procedure, organ and space surgical site |
|  | L03 | Cellulitis/abscess of skin, various locations |
|  | L08 | Other local infections of skin and subcutaneous tissue |
|  | L92 | Granulomatous conditions of skin/subcut. tissue, other disorders of skin/subcut. tissue |
|  | L98 | Other disorders of skin and subcutaneous tissue, not elsewhere classified |
|  | T8149 | Infection following a procedure, other surgical site |
| **Sepsis** | A40 | Streptococcal sepsis |
|  | A41 | Other sepsis |
|  | R6510 | Systemic inflammatory response syndrome (SIRS) of non-infectious origin without acute organ dysfunction |
|  | R6511 | Systemic inflammatory response syndrome (SIRS) of non-infectious origin with acute organ dysfunction |
|  | R6520 | Severe sepsis without septic shock |
|  | T8144 | Sepsis following a procedure |
|  | R6521 | Severe sepsis with septic shock |
| **Anastomotic leak** | K9189 | Other postprocedural complications and disorders of digestive system, including anastomotic leak |
| **Seroma** | L7634 | Postprocedural seroma of skin and subcutaneous tissue following other procedure |
| **Nausea/vomiting** | K910 | Vomiting following gastrointestinal surgery |
|  | K920 | Hematemesis |
|  | R110 | Nausea |
|  | R1110 | Vomiting, unspecified |
|  | R1111 | Vomiting without nausea |
|  | R112 | Nausea with vomiting, unspecified |
|  | R1113 | Vomiting of fecal matter |
| **Nutrient Malabsorption** | K912 | Postsurgical malabsorption, not elsewhere classified |
|  | K909 | Intestinal malabsorption, unspecified |
|  | E46 | Unspecified protein-calorie malnutrition |
|  | E440 | Moderate protein-calorie malnutrition |
|  | E441 | Mild protein-calorie malnutrition |
|  | E43 | Unspecified severe protein-calorie malnutrition |
| **Mallory-Weiss syndrome** | K226 | Gastro-esophageal laceration-hemorrhage syndrome (Mallory-Weiss syndrome) |
| **Bowel obstruction** | K315 | Obstruction of duodenum |
|  | K565 | Intestinal adhesions [bands] with obstruction (postinfection) |
|  | K566 | Other and unspecified intestinal obstruction |
|  | K5669 | Other intestinal obstruction |
|  | K562 | Volvulus |
|  | K5641 | Fecal impaction |
|  | K5649 | Other impaction of intestine |
|  | K913 | Postprocedural intestinal obstruction |
| **Incisional hernia** | K430 | Incisional hernia with obstruction, without gangrene |
|  | K431 | Incisional hernia with gangrene |
|  | K432 | Incisional hernia without obstruction or gangrene |
| **Acute perforation or ulcer** | K9171 | Accidental puncture and laceration of a digestive system organ or structure during a digestive system procedure |
|  | K9172 | Accidental puncture and laceration of a digestive system organ or structure during other procedure |
|  | K250 | Acute gastric ulcer with hemorrhage |
|  | K253 | Acute gastric ulcer without hemorrhage or perforation |
|  | K251 | Acute gastric ulcer with perforation |
|  | K252 | Acute gastric ulcer with both hemorrhage and perforation |
|  | K260 | Acute duodenal ulcer with hemorrhage |
|  | K261 | Acute duodenal ulcer with perforation |
|  | K262 | Acute duodenal ulcer with both hemorrhage and perforation |
|  | K263 | Acute duodenal ulcer without hemorrhage or perforation |
|  | K271 | Acute peptic ulcer, site unspecified, with perforation |
|  | K272 | Acute peptic ulcer, site unspecified, with both hemorrhage and perforation |
|  | K281 | Acute gastrojejunal ulcer with perforation |
|  | K282 | Acute gastrojejunal ulcer with both hemorrhage and perforation |
|  | K280 | Acute gastrojejunal ulcer with hemorrhage |
|  | K283 | Acute gastrojejunal ulcer without hemorrhage or perforation |
|  | K289 | Gastrojejunal ulcer, unspecified as acute or chronic, without hemorrhage or perforation |
|  | E3602 | Intraoperative hemorrhage and hematoma of an endocrine system organ or structure complicating other procedure |
|  | E3612 | Accidental puncture and laceration of an endocrine system organ or structure during other procedure |
|  | E89811 | Postprocedural hemorrhage of an endocrine system organ or structure following other procedure |
|  | E89821 | Postprocedural hematoma of an endocrine system organ or structure following other procedure |
|  | K9161 | Intraoperative hemorrhage and hematoma of a digestive system organ or structure complicating a digestive system procedure |
|  | K9162 | Intraoperative hemorrhage and hematoma of a digestive system organ or structure complicating other procedure |
| **Venous thromboembolism** | I801 | Phlebitis and thrombophlebitis of femoral vein |
|  | I802 | Phlebitis and thrombophlebitis of other and unspecified deep vessels of lower extremities |
|  | I26 | Pulmonary embolism |
|  | I803 | Phlebitis and thrombophlebitis of lower extremities, unspecified |
| Note: ICD codes were identified using partial string matching. Any code that contains the character sequence shown (including all descendant/child codes that share that prefix) was included. | | |
